# Functional diversity of 2-oxoglutarate/Fe(II)-dependent dioxygenases in plant metabolism

**DOI:** 10.3389/fpls.2014.00524

**Published:** 2014-10-09

**Authors:** Scott C. Farrow, Peter J. Facchini

**Affiliations:** Department of Biological Sciences, University of CalgaryCalgary, AB, Canada

**Keywords:** 2-oxoglutarate/Fe(II)-dependent dioxygenase, primary metabolism, specialized metabolism, DNA and histone demethylation, iron sensing

## Abstract

Oxidative enzymes catalyze many different reactions in plant metabolism. Among this suite of enzymes are the 2-oxoglutarate/Fe(II)-dependent dioxygenases (2-ODDs). Cytochromes P450 (CYPs) as often considered the most versatile oxidative enzymes in nature, but the diversity and complexity of reactions catalyzed by 2-ODDs is superior to the CYPs. The list of oxidative reactions catalyzed by 2-ODDs includes hydroxylations, demethylations, desaturations, ring closure, ring cleavage, epimerization, rearrangement, halogenation, and demethylenation. Furthermore, recent work, including the discovery of 2-ODDs involved in epigenetic regulation, and others catalyzing several characteristic steps in specialized metabolic pathways, support the argument that 2-ODDs are among the most versatile and important oxidizing biological catalysts. In this review, we survey and summarize the pertinent literature with a focus on several key reactions catalyzed by 2-ODDs, and discuss the significance and impact of these enzymes in plant metabolism.

## Introduction

The first reported dioxygenase activity was pyrocatechase from *Pseudomanas* spp., which catalyzes the 1,2-intradiol cleavage of pyrocatechin to muconic acid (Hayaishi and Hashimoto, 1950). In this reaction, both atoms of molecular oxygen are incorporated into the muconic acid product, leading to use of the term “di”oxygenase (Hayaishi et al., 1956). By definition, dioxygenases have the ability to incorporate two atoms of molecular oxygen into one or more substrates. However, this definition can be ambiguous owing to the number of different dioxygenase types, which vary by the nature of their co-factor and co-substrate dependence. This review is focused on the mononuclear iron dioxygenases, with particular emphasis on a specific sub-class, the 2-oxoglutarate/Fe(II)-dependent dioxygenases (2-ODDs).

**Scheme 1:**
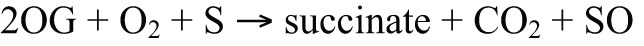
**Catalytic mechanism for 2-oxoglutarate-dependent dioxygenases**. Abbreviations: S, prime substrate; SO, oxidized prime substrate.

2-ODDs are non-heme proteins belonging to a large superfamily that are ubiquitously distributed throughout nature, and occur in bacteria, fungi, plants, and vertebrates (De Carolis and De Luca, 1994; Martens et al., 2010). The landmark discovery of the first 2-ODD revealed its hydroxylation capacity for prolyl and lysyl amino acid residues, and established the necessary factors required for enzyme function (Hutton et al., 1967). 2-ODDs are dependent on ferrous iron as a co-factor for the binding of molecular oxygen and subsequent oxidative reactions. In essentially all cases, 2-ODDs couple the two-electron oxidation of the prime substrate [S] to the oxidative decarboxylation of the distinctive co-substrate—2-oxoglutarate (2OG)—giving rise to succinate and carbon dioxide (Scheme 1; Wilmouth et al., 2002; Zhang et al., 2004; Flashman and Schofield, 2007; Hangasky et al., 2013). The oxidation of the prime (S) substrate leads to the formation of specific products (SO).

In addition to 2OG and ferrous iron, 2-ODDs activity is usually increased by the addition of catalase and ascorbate. Catalase serves as a protecting agent from hydrogen peroxide (Prescott and John, 1996), whereas ascorbate—although not always essential—supports enzyme function by completing spontaneous-uncoupled reactions (Clifton et al., 2006) and is thought to assist with enzymatic cycles by maintaining the ferrous iron state (De Carolis and De Luca, 1994; Prescott and John, 1996).

2-ODDs facilitate numerous oxidative reactions including hydroxylations, halogenations, desaturations, epimerization, cyclizations, and ring formation, ring fragmentation, C-C bond cleavage, re-arrangements, demethylations, and demethylenations (Clifton et al., 2006; Flashman and Schofield, 2007; Loenarz and Schofield, 2008; Tarhonskaya et al., 2014). This impressive list of reactions reveals the versatility of these enzymes in catalyzing many reactions that are still not possible using synthetic chemistry (Flashman and Schofield, 2007). The significance of 2-ODDs is underscored by their widespread roles in biosynthetic pathways essential for normal organismal function, or that lead to high-valued specialized metabolites.

The *Arabidopsis thaliana* genome contains more than 130 2-ODD genes, representing approximately 0.5% of the total gene complement (Kawai et al., 2014). However, only a handful of plant 2-ODDs have been functionally characterized. Extrapolating from the number of 2-ODD genes in Arabidopsis, a plethora of reactions and roles for 2-ODDs in other plant species can be predicted. Given the known importance of 2-ODDs in plant metabolism, the continued functional characterization of 2-ODDs is essential. Here we describe known biochemical functions for plant 2-ODDs, with a focus on recent discoveries and the impact of 2-ODDs on plant metabolism.

## Structural and mechanistic features of 2-oxoglutarate/FE(II)-dependent dioxygenases

**Figure 1:**
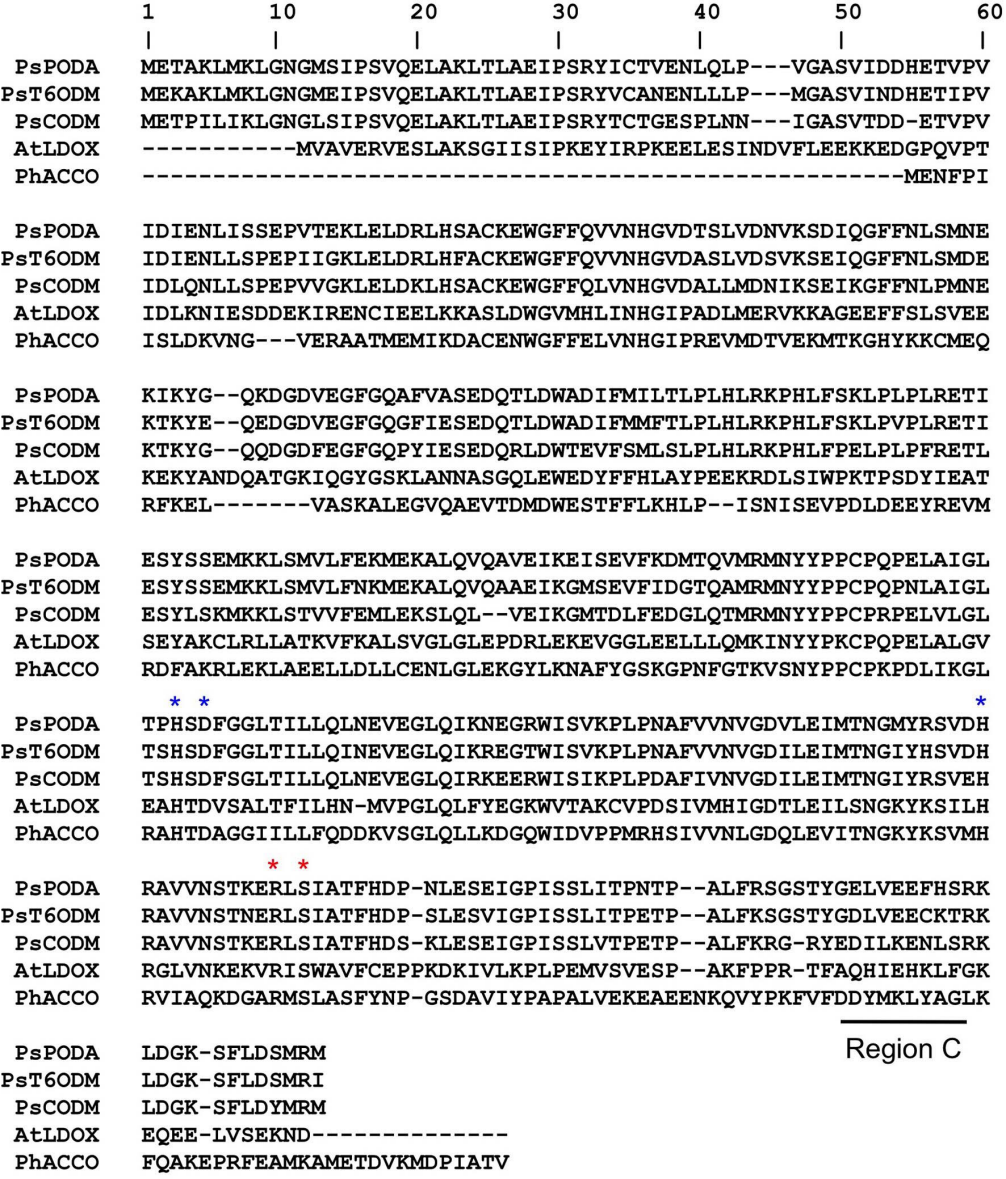
**Sequence alignments of *O-demethylases* from *Papaver somniferum* and 2-ODDs from other plants for crystal structures have been determined**. PsCODM, codeine *O-demethylase*; PsT6ODM, thebaine 6-*O*-demethylase; PsPODA, protopine *O*-dealkylase; AtLDOX, leucoanthocyanidin dioxygenase; PhACCO, 1-aminocyclopropane-1-carboxylic acid oxidase. Blue asterisks indicate a conserved catalytic triad motif (H_X_D_Xn_H). Red asterisks indicate proposed 2OG binding residues. Region C contains residues associated with the regiospecific 3-*O*-demethylation of thebaine (Runguphan et al., 2012). Comprehensive alignments and phylogenetic trees of numerous plant 2-ODDs have been reported (Kawai et al., 2014).

Structural analyses of leucoanthocyanidin synthase (LDOX; Wilmouth et al., 2002; PDB:1GP4), 1-aminocyclopropane-1-carboxylic acid oxidase (ACCO; Zhang et al., 2004; PDB:1W9Y) and several related 2-ODDs have revealed canonical structural features, including a double stranded β-helix core fold known as jellyroll topology that supports and protects a catalytic triad of Fe(II) binding residues (Clifton et al., 2006). These residues are comprised of a highly conserved, but not ubiquitous, HX(D/E)X_n_H triad motif that is essential for binding Fe(II) (Figure 1). Residues conferring 2OG binding are less conserved and are usually characteristic of 2-ODD sub-families, whereas those linked to binding of the prime substrate are variable, but might involve features in proximity to the active core (Loenarz and Schofield, 2008).

**Figure 2:**
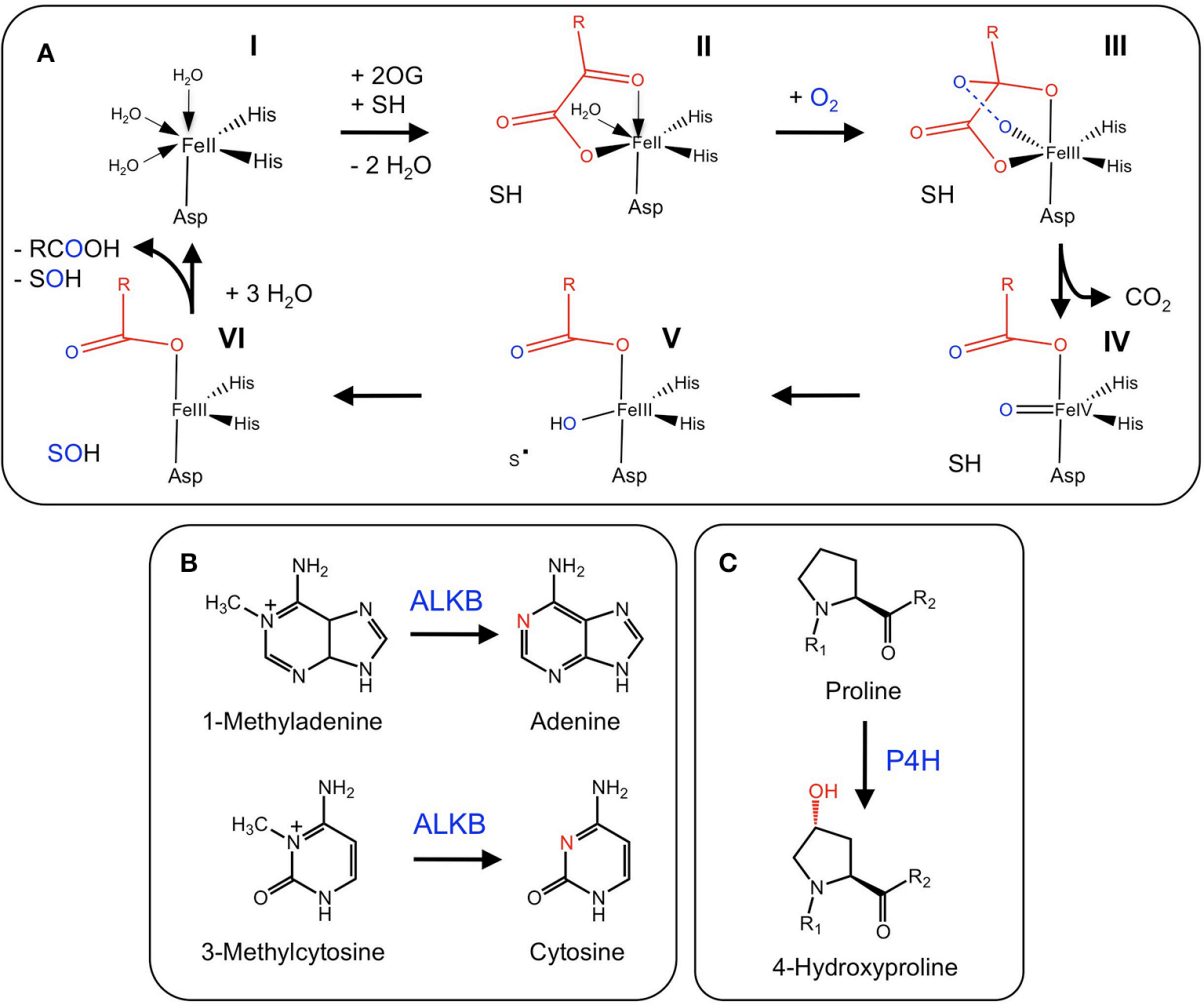
**(A)** Proposed mechanisms of 2-oxoglutarate binding and formation of the reactive iron-oxo intermediate for the oxidation of the prime substrate. (I) Ferrous iron binds to the active site of the jellyroll structure. (II) Addition of 2-oxoglutarate displaces two water molecules and permits the bidentate binding of 2-oxoglutarate. Subsequent binding of the prime substrate (SH) in (II) vacates a third binding site for molecular oxygen (III). Oxidative decarboxylation of 2-oxoglutarate (III-IV) yields the reactive iron-oxo intermediate and succinate. (IV-VI) Hydrogen abstraction and two-electron oxidation of the prime substrate leads to the formation of the product (SOH) and the release of succinate for subsequent enzyme cycles (Adapted from Tarhonskaya et al., 2014). **(B)** DNA repair via the demethylation of N_6_ and N_1_ of adenine and cytosine, respectively, by ALKB. **(C)** P4H hydroxylates proline residues leading to the formation of 4-hydroxyproline. P4H is involved in the post-translational modification of structural proteins. A subclass of these proteins (HIF) is involved in oxygen sensing in mammals, whereas and iron-sensing role has been proposed for the equivalent homologs in plants.

Detailed crystallographic, spectroscopic and kinetic analyses have revealed mechanistic features that are apparently ubiquitous for the formation of the high-valent iron-oxo intermediate responsible for oxidation of the prime substrate. The consensus mechanism involves binding of ferrous iron, which displaces two water molecules and permits the bidentate binding of 2OG (C-1 carboxyl, C-2 keto). Binding of the prime substrate weakens an additional water molecule and exposes another iron binding site for molecular oxygen. Subsequent decarboxylation of 2OG gives rise to CO_2_ and a succinate bound iron-oxo intermediate. The iron-oxo intermediate is essential for oxidation of the prime substrate (Figure 2A), and has been likened to Fenton chemistry (Prescott and John, 1996; Groves, 2006). Oxidative mechanisms are specific to each prime substrate, and several different mechanisms have been proposed (Wilmouth et al., 2002). Some atypical 2-ODDs, such as an enzyme involved in ethylene formation, have a 2OG binding motif, but utilize ascorbate instead of 2OG for formation of the reactive iron-oxo intermediate (See below for details of this mechanism; Hausinger, 2004).

## 2-ODDs in primary metabolic networks

### DNA repair

Alkylating agents are ubiquitous throughout nature and are a significant contributor to RNA and DNA damage, as well as tumorigenesis (Mielecki et al., 2012). In depth analysis of their target substrates has revealed a capacity for nucleic acid alkylation, which leads to toxic, mutagenic or neutral chemical derivatives (Drabløs et al., 2004). As a means to counter these potentially deleterious chemicals, several organisms have evolved repair mechanisms. One mechanism has been described for *Escherichia coli* ALKB proteins, which are 2-ODDs capable of repairing a number of modifications to the N^1^ and N^3^ positions of adenine and cytosine, respectively (Lindahl et al., 1988; Mielecki et al., 2012). Until recently, no active ALKB homologs were known from plants. However, a homolog of human *alkbh2* and *alkbh3* has been identified in *Arabidopsis thaliana* (Meza et al., 2012). Recombinant AtALKBH2 prefers *ds*DNA over *ss*DNA, and is capable of reversing etheno lesions, which is also a function of human ALKBH2 (Figure 2B). Furthermore, *Arabidopsis thaliana* mutants with a defective *alkbh2* were sensitive to the methylating agent methylmethanesulphonate, which supports a role for ALKBH2 in the removal of deleterious methyl lesions from plant genomes. Sequence and functional similarities between human and plant ALKBH2 suggests that these proteins are true orthologs (Meza et al., 2012).

Sequence similarity among AtALKB homologs is insufficient for the assignment of protein function. Although AT3G14160 (NP_566479.5), and AT1G11780 (NP_172643.1) display the highest degree of sequence similarity to the characterized and functional *E. coli* ALKB, these Arabidopsis proteins did not show DNA repair activity in phage reactivation assays (Meza et al., 2012). The same observation is true for human ALKBH1, which shows high homology with functional *E. coli* ALKB, yet only ALKBH2 and ALKBH3 possess DNA repair activities (Mielecki et al., 2012). Other ALKB homologs from human and Arabidopsis appear to have different functions (Meza et al., 2012).

### Histone demethylation

Histone methylation plays a fundamental role in gene activation or repression in eukaryotic species (Cho et al., 2012). Although the covalent modification of histone residues is usually reversible, the reversibility of methylations was questionable (Tsukada et al., 2006) until the landmark discovery of the first histone demethylase (Shi et al., 2004). The reversible methylation and demethylation of histone residues are key components in the epigenetic modulation of gene expression. Several histone demethylases have been identified and characterized from numerous eukaryotes, and these are categorized into two main categories: (i) flavin adenine dinucleotide oxidase demethylases and (ii) 2-ODD demethylases.

Characterization of plant homologs representing both families has revealed numerous physiological roles including the regulation of flowering time (Jeong et al., 2009; Lu et al., 2010; Yang et al., 2010), de-etiolation (Charron et al., 2009), female gametophyte development (Pagnussat et al., 2005), brassinosteroid signaling (Yu et al., 2008), floral organ development (Sun and Zhou, 2008) and circadian regulation (For a review see: Jones et al., 2010; Chen et al., 2011; Lu et al., 2011). Critical among these enzymes are the “Jumonji” (Takeuchi et al., 1995) class of 2-ODD demethylases (JmjC).

Recently, two *JmjC* genes, *Jmj20* and *Jmj22*, encoding 2-ODDs were linked to seed development in Arabidopsis. Interestingly, *Jmj20* and *Jmj22* are induced by light via the activation of cytochrome B. Jmj20 and Jmj22 act redundantly to reverse repressive histone methylation, which allows transcription of key gibberellin biosynthetic genes, gibberellin 3-oxidase and gibberellin 2-oxidase. The subsequent increase in the levels of active gibberellins stimulates seed germination (Cho et al., 2012).

Another 2-ODD, Jmj703, was linked to plant cell division and stem elongation via the demethylation of mono-, di-, and tri-methylated lysine-4 residues of histone subunit 3 (Chen et al., 2013). Interestingly, loss-of-function mutants displayed a dwarf phenotype and cytokinin oxidase genes (CKX), which control the deactivation of cytokinin, were expressed at elevated levels compared with wild type plants. Additionally, the methylation status of the promoter region of *CKX* genes was increased in mutant plants, suggesting that Jmj703 plays a role in regulating the expression of *CKX* genes and the proper plant growth and development via the modulation of active cytokinin pools.

### Post-translational modification and iron sensing

Post-translational modifications (PTMs) are one of several methods whereby organisms extend or alter protein function. One of the most abundant PTMs is proline hydroxylation, which is catalyzed by the 2-ODD, proly 4-hydroxylase (P4H) (Gorres and Raines, 2010). The product of P4H, 4-hydroxyproline, occurs in numerous proteins in animals and is found in different plant glycoproteins (Soares et al., 2011). In animals, 4-hydroxyproline confers structural integrity to collagen. Proline PTMs also occur in several structural proteins and are essential for normal plant growth and development. Although many P4Hs have been characterized from animals (Myllyharju, 2003), the biochemical functions of only two Arabidopsis P4Hs have been determined (Hieta and Myllyharju, 2002; Tiainen et al., 2005). Arabidopsis P4Hs exhibit proline 4-hydroxylation activity, but show a variable substrate range and kinetic properties suggesting that each protein possesses a unique function (Figure 2C). Although authentic targets have not been identified, Arabidopsis *P4H* genes are highly expressed in roots and display differential responses to various stresses (Vigani et al., 2013).

The hypoxia-inducible transcription factor (HIF) is regulated by a P4H subclass (HIF-P4H). Under normoxic conditions, the HIFα sub-unit is constitutively synthesized and contains a critical 4-hydroxy proline residue. The presence of 4-hydroxyproline is essential for rapid and continuous proteasomal degradation. Alternatively, under hypoxic conditions, HIF-P4H is inactive and the proline residue is no longer modified. Consequently, HIFα is salvaged and can form a stable dimer with the HIFβ subunit. The dimer is translocated into the nucleus where it docks with HIF-responsive elements, which activates a cascade of hypoxia-responsive genes. As such, HIF-P4Hs are thought to be key oxygen sensors in animals (Vigani et al., 2013).

In plants, the functions of HIF-P4H homologs are not well understood. One hypothesis is that HIF-P4Hs in plants modulate metabolic changes during iron starvation. Interestingly, several of these changes involve the induction of 2-ODDs with roles in the reutilization of iron (Jin et al., 2007; Lan et al., 2011) or the scavenging of iron from the soil (Kobayashi et al., 2001). Moreover, HIF-P4Hs have a relatively low *K*_m_ for iron (Hieta and Myllyharju, 2002; Tiainen et al., 2005). Whereas animal HIF-P4Hs sense oxygen, plant homologs are thought to function as iron sensors by monitoring the availability of iron. Taken together, a link might exist between specific 2-ODDs, such as P4H, and iron deficiency responses via some specific metabolic reprogramming. Such advancements in the field of plant nutrient sensing and signaling could contribute to solutions addressing several global agricultural challenges (Vigani et al., 2013).

### Gibberellin biosynthesis and catabolism

**Figure 3:**
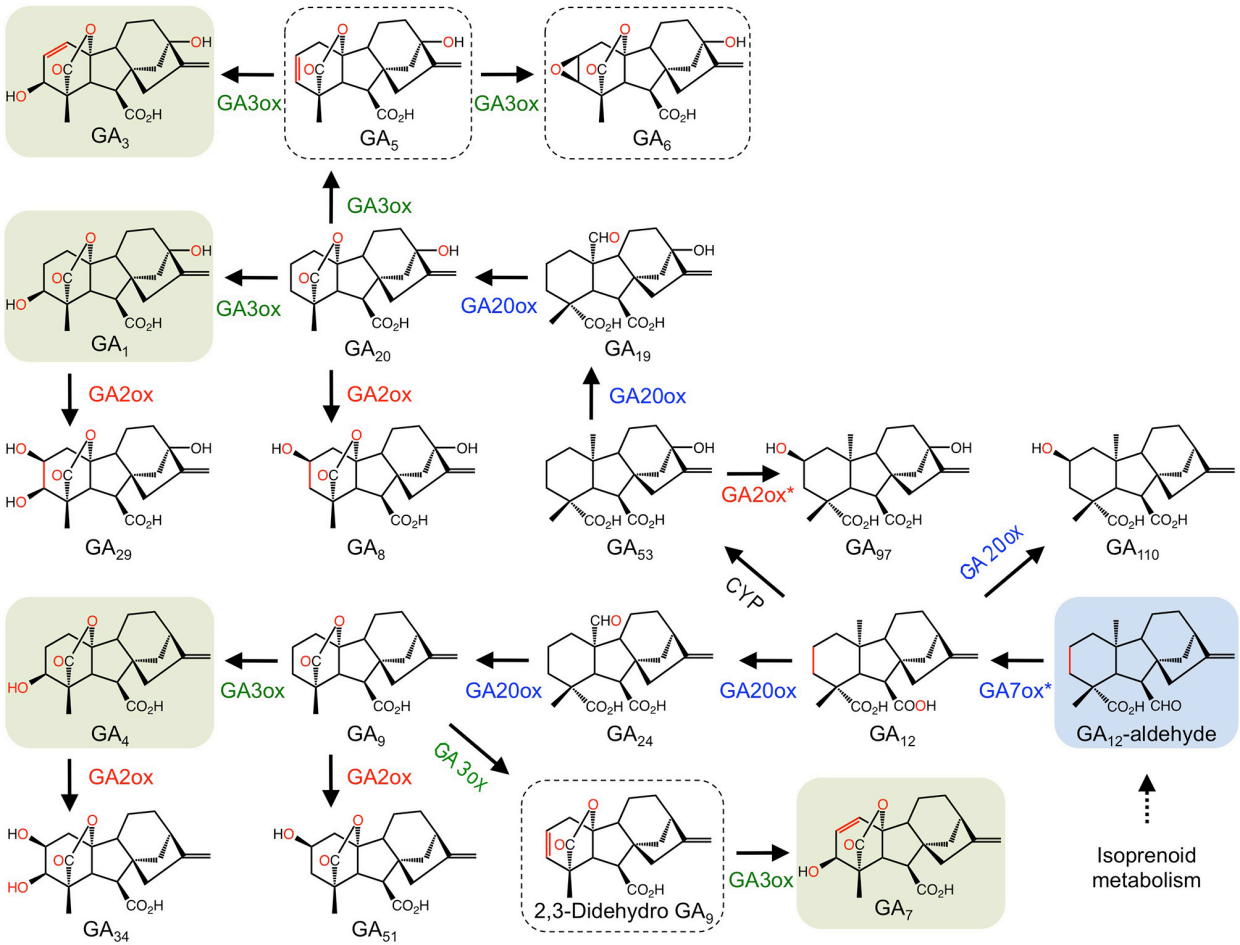
**Participation of 2-ODDs in gibberellin metabolism**. Blue labels are enzymes involved in major routes to active GAs. Red and green labels are enzymes responsible for GA deactivation and activation, respectively. Green boxes show active GAs. Hashed boxes represent GAs with suspected bioactivity (Yamaguchi, 2008). The blue box highlights the central intermediate to all GAs. The asterisk refers to a step catalyzed by a 2-ODD in *Cucurbita maxima*, but catalyzed by a CYP in other species (Adapted from: Yamaguchi, 2008).

Gibberellins (GAs) are a group of diterpene-derived plant growth regulators that play a key role in many growth and developmental processes including stem elongation, leaf expansion, flower development, and germination (MacMillan, 2001). The well-defined biosynthesis of GAs includes several 2-ODDs that are responsible for several structural modifications (Yamaguchi, 2008) (Figure 3). The 2-ODD GA 20-oxidase (GA20ox) catalyzes the three-step oxidation of inactive GA_12_ or GA_53_ to the immediate precursors of active GAs (Lange et al., 1994a; Xu et al., 1995; Lange, 1997). Activation of the GA scaffold is performed by other 2-ODDs, GA 3-oxidases (GA3ox), which shows strict regio-specificity for the C-3 position of GA_5_, GA_9_, and GA_20_ (Chiang et al., 1995; Lange et al., 1997). In contrast, GA 2-oxidases (GA2ox) play a role in GAs catabolism via C-2 hydroxylation of the GA backbone (Thomas et al., 1999; Schomburg et al., 2003; Lee and Zeevaart, 2005). Interestingly, pumpkin (*Cucurbita maxima L*.) contains one additional enzyme, GA7 oxidase (GA7ox), which catalyzes the C-7 oxidation of GA_12_ aldehyde to produce the carboxylic acid moiety of GA_15_. The occurrence of this enzyme in pumpkin is unusual because the same reaction is also catalyzed by a highly conserved CYP (CYP88A) (Hedden et al., 2002). The occurrence of functionally redundant enzymes in pumpkin might reflect the occurrence of disparate and isolated GA pathways leading to different compounds (Lange et al., 1994b).

GA metabolism in plants and fungi is notably different at later metabolic stages. In fungi, CYPs are the prominent enzyme type catalyzing the biosynthesis of active GAs, whereas plants predominantly use 2-ODDs (Hedden et al., 2002). Occasionally, plants and fungi utilize both enzyme types (Lange et al., 1994b).

### Ethylene biosynthesis

**Figure 4:**
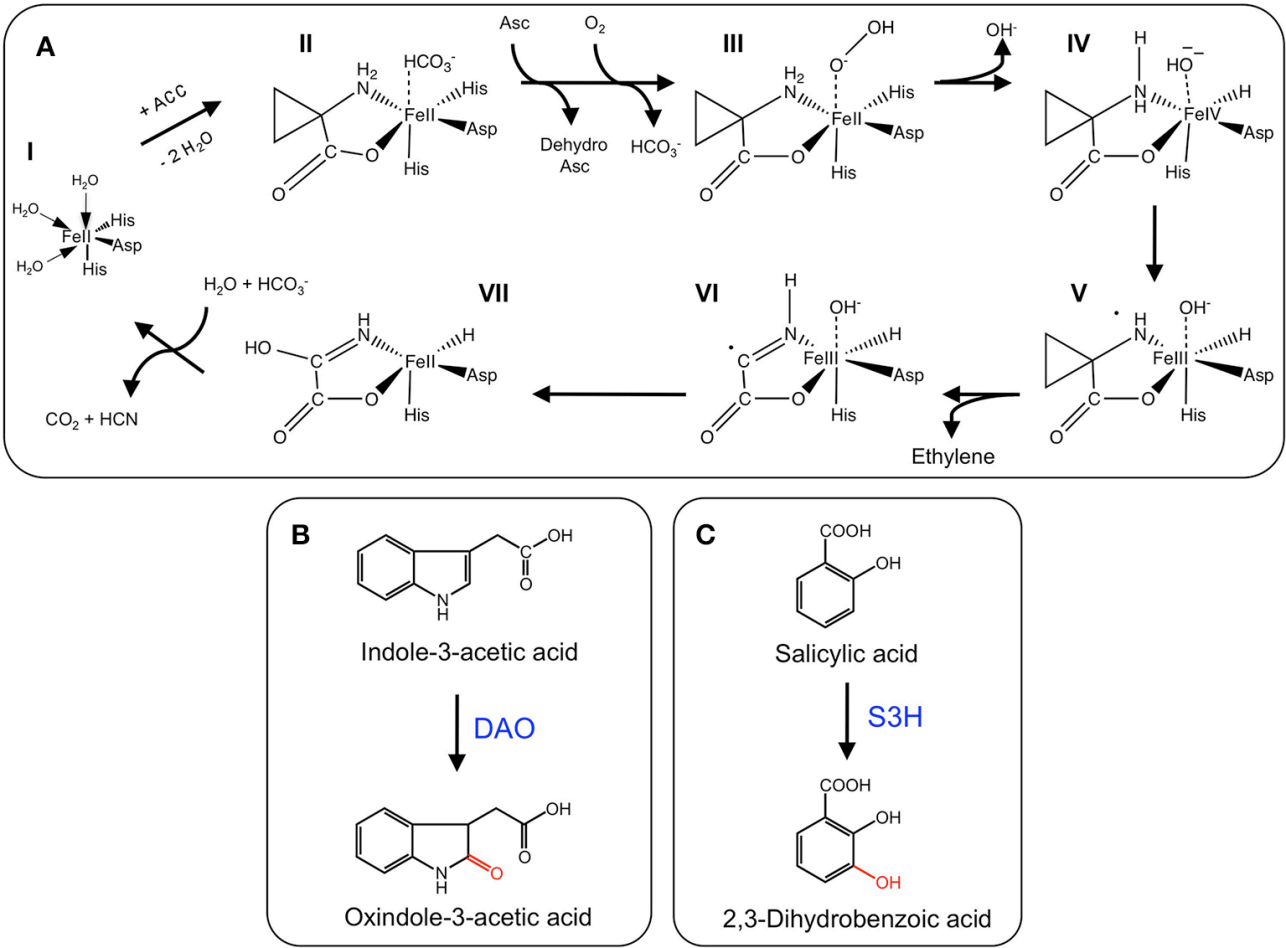
**(A)** Proposed mechanism of ethylene formation by ACCO. (I) Ferrous-iron binding in the active site of the jellyroll structure. (II) Addition of ACC displaces two water molecules and permits binding. CO_2_ maximizes enzyme activity by reacting with a proposed metal bound solvent molecule to generate a HCO^−^_3_ bound species. (III) Addition of ascorbate stimulates the loss of the bound HCO^−^_3_ species and enhances reactivity with molecular oxygen. Ascorbate donates two electrons to generate the ion-oxo intermediate. (IV) Loss of hydroxide (hydrolytic cleavage) and formation of the iron-oxo intermediate. (V) Hydrogen atom abstraction (IV-V), rearrangement (V-VI), and hydroxyl radical transfer (VI-VII) form the metal-bound intermediate that decomposes to CO_2_ and hydrogen cyanide (VII-I) (Adapted from Hausinger, 2004). **(B)** Oxidation of IAA by DAO. **(C)**. Hydroxylation of salicylic acid by S3H. S3H.

The gaseous plant growth regulator ethylene has roles in several plant growth and developmental processes including senescence and fruit ripening. Ethylene biosynthesis begins with the conversion of L-methionine to *S*-adenosyl-L-methionine (SAM) by SAM synthetase. SAM is converted to the immediate precursor to ethylene, 1- aminocyclopropane-1-carboxylic acid (ACC), by ACC synthase. The final step is catalyzed by the 2-ODD, ACC oxidase (ACCO; Figure 4A). Since the initial isolation of ACCO cDNAs (Nakajima and Mori, 1990; Van der Straeten et al., 1990), native and recombinant proteins have been characterized from several plants (Kende, 1993). An interesting feature of the ACCO reaction is the role of carbon dioxide in preventing auto-inactivation of the enzyme. Furthermore, despite having the conserved binding motif, ACCO does not utilize 2OG as a substrate. Instead, ascorbic acid acts as a two-electron reductant critical to the catalytic mechanism (Hausinger, 2004). The generally accepted mechanistic model for ACCO involves the formation of bicarbonate, which is thought to occur via reaction of CO_2_ with an iron bound molecule. Binding of 1- aminocyclopropane-1-carboxylic acid followed by oxygen activation via ascorbate-mediated bicarbonate reduction leads to the formation of the iron-oxo intermediate, which subsequently oxidizes 1-aminocyclopropane-1-carboxylic acid leading to the production of ethylene, hydrogen cyanide, and carbon dioxide (Figure 4A).

### Auxin catabolism

Indole-3-acetic acid or auxin (IAA) is a plant growth regulator with many key roles in plant growth and development. As such, IAA biosynthesis, transport, and signaling are highly regulated processes. The general model for IAA regulation involves a network of synthesis, degradation, conjugation, and transport (Woodward and Bartel, 2005). Despite knowledge of numerous IAA biosynthetic and conjugative enzymes, and an understanding of several transport mechanisms, the catabolism and degradation of IAA are not as well understood. Recently, a 2-ODD (DAO) from rice was shown to catalyze the irreversible inactivation of IAA to its oxindole derivative (OxIAA) (Figure 4B). The *dao* gene was linked with IAA catabolism by characterizing the *dao* mutant, which showed significantly higher levels of active IAA, and an apparent lack of OxIAA. Mutant *dao* plants also displayed several developmental abnormalities to male reproductive organs and were infertile. Moreover, unfertilized ovaries produced parthenocarpic seeds. As such, it was suggested that *dao* assists in the regulation of active IAA pools in rice flowers with consequences on the development of male organs (Zhao et al., 2013).

### Salicylic acid catabolism

The phenolic plant growth regulator salicylic acid (SA) is involved in several plant growth and developmental processes including plant defense, stress responses, and senescence (Zhang et al., 2013). Although SA biosynthesis is well established, SA catabolism is not well understood. Recently, the major inactive forms of SA in senescing leaves were identified as 2,3 and 2,5-dihydroxybenzoic acid sugar conjugates (2,3-DHBA and 2,5-DHBA, respectively), suggesting that hydroxylation is involved in SA catabolism (Bartsch et al., 2010). The At4g10500 gene from Arabidopsis is a 2-ODD annotating as a senescence-related gene. Interestingly, At4g10500 transcripts accumulate during senescence and upon treatment with SA. Using a T-DNA insertion line, At4g10500 transcripts were abolished, which dramatically increased the rate of leaf senescence. Further characterization of the T-DNA knockout line, an over-expression line, and wild type plants revealed striking differences to the levels of active SA and derivatives. In particular, active SA levels increased dramatically in the T-DNA line compared with both over-expression and wild type lines. In contrast, the major inactive sugar catabolites of 2,3-DHBA were undetectable in the knockout line, but accumulated to approximately 200% of wild type levels in the over-expression line. These data implicate At4g10500 as the gene encoding salicylic acid 3-hydroxylase (S3H), which is responsible for the inactivation of SA to 2,3-DHBA. Recombinant S3H converted SA (*K*_m_ = 58.29 μ M) to both 2,3-dihydroxybenzoic acid (2,3-DHBA) and 2,5-DHBA (Figure 4C), in support of a role for S3H in the modulation of active SA levels by converting SA to 2,3-DHBA. The irreversible conversion of SA to 2,3-DHBA allows conjugation via the addition of a sugar moiety, which yields the major inactive SA in senescing leaves (Zhang et al., 2013).

## 2-ODDs in secondary metabolism

### Flavonoid biosynthesis and metabolism

Flavonoids are a highly diverse class of specialized metabolites widely distributed in higher plants, and also found in liverworts and horsetails (Martens et al., 2010). Among the numerous functions assigned to flavonoids are protection from UV-B irradiation (Li et al., 1993), signaling with insects and plants (Harborne and Williams, 2000), and a general adaptation to ecological niches during periods of abiotic stress (Martens et al., 2010). Some flavonoids also exhibit pharmacological properties including sedative (Fernández et al., 2004) and anti-inflammatory effects (Williams et al., 1999; Harsteen, 2002).

Flavonoid biosynthesis begins with the amino acid phenylalanine, which undergoes deamination to *trans*-cinnamic acid. Subsequent oxidation of *trans*-cinnamic acid yields *p*-coumaric acid, which undergoes transformation to *p*-coumaroyl-CoA. The committed step in flavonoid biosynthesis is catalyzed by chalcone synthase, which yields chalcone via the condensation of one molecule of *p*-coumaroyl-CoA and three molecules of malonyl-CoA. Subsequent isomerization of chalcone generates the tricyclic (*2S*)-flavonone backbone (Shirley, 1996), which is subsequently modified via different enzymes, including glucosyltransferases, *O*-methyltransferases, CYPs and 2-ODDs. These modifications lead to the diversity of flavonoids found in plants, including isofavones, flavones, flavonols, anthocyanidins, and proanthocyanidins.

**Figure 5:**
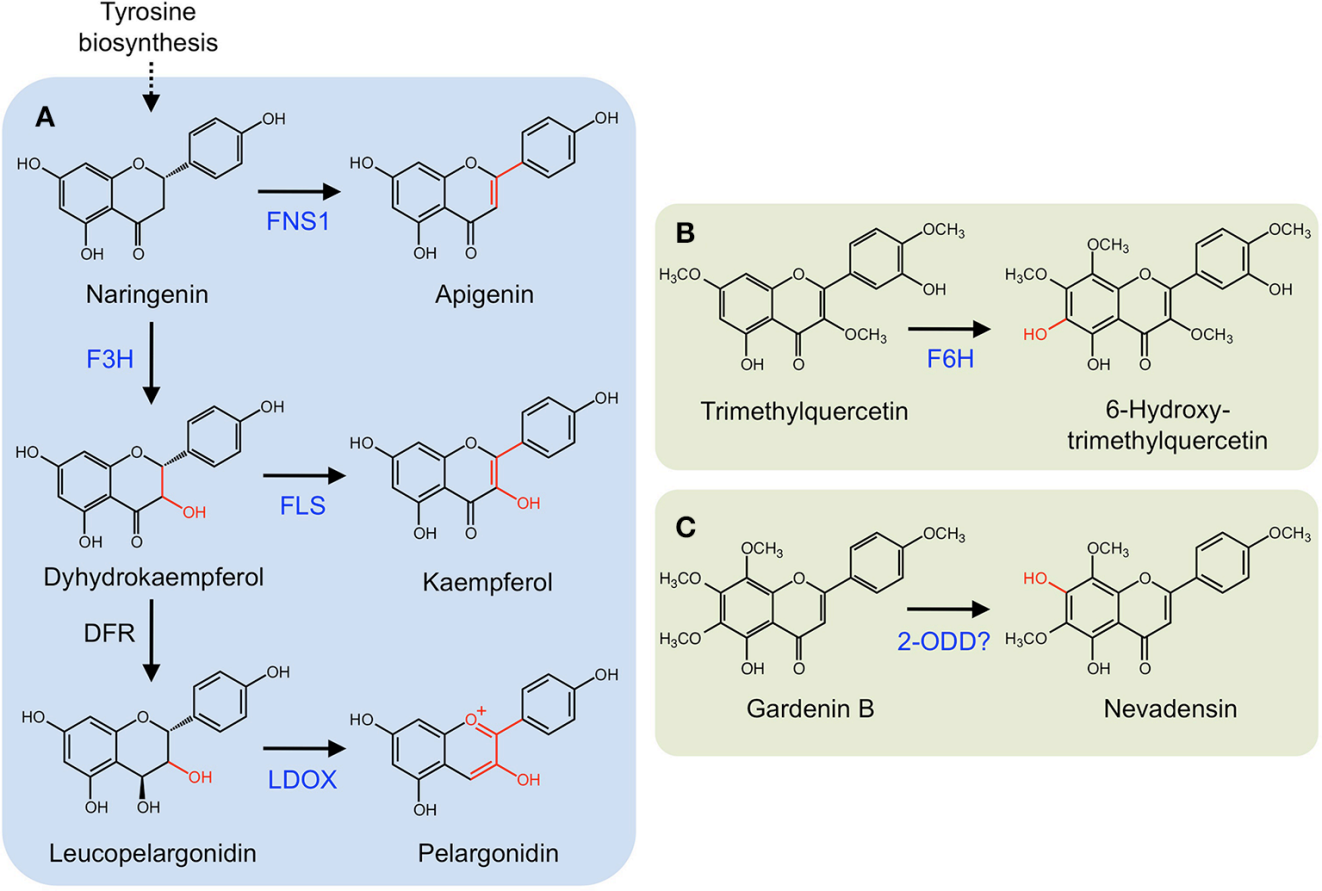
**(A)** Core flavonoid metabolism. Oxidation of (*2S*)-flavonones by FNS I. (*2S*)-Flavonones can also be converted to dihydroflavonols by F3H. Dihydroflavonols serve as substrates for flavonol synthase (FLS), competing with dihydroflavonol 4-reductase (D4R) and leucoanthocyanidin synthase (LDOX). **(B)** 6-Hydroxy-trimethylquercetin formation by F6H. **(C)** *O*-Demethylation of gardenin B to nevadensin.

Several 2-ODDs catalyze key oxidative reactions that facilitate the formation of different flavonoid subclasses (Figure 5A). Oxidation of (*2S*)-flavonones, by the 2-ODD flavone synthase (FNS I), introduces a double bond at C-2/C-3 to yield flavones. FNS I occurs primarily in members of the Apiaceae (Britsch, 1990; Martens et al., 2001; Gebhardt et al., 2005, 2007), but has also been reported in *Oryza sativa* (Lee et al., 2008) and *Equisetum arvense* (Bredebach et al., 2011). Alternatively, a CYP (FNS II) is thought to catalyze the same reaction in larger number of plants (Kochs and Grisebach, 1987; Akashi et al., 1999; Martens and Forkmann, 1999; Davies and Schwinn, 2007). (*2S*)-Flavonones are converted to dihydroflavonols by C-3 hydroxylation, which is catalyzed by another 2-ODD, flavonone 3β-hydroxylase (F3H; Britsch and Grisebach, 1986; Britsch et al., 1992). The location of F3H in the biosynthetic pathway has implications for the type and quantity of downstream products since it competes with FNS I and FNS II for available (*2S*)-flavonones (Martens et al., 2010). Dihydroflavonols serve as substrates for flavonol synthase (FLS), which competes with dihydroflavonol 4-reductase (D4R) and LDOX for the production of flavonols or anthocyanidins and proanthocyanidins, respectively. FLS and ANS are also 2-ODDs. FLS introduces a double bond at C-2/C-3 to yield flavonols (Spribille and Forkmann, 1984; Holton et al., 1993), whereas D4R selectively reduces the C-4 keto group of dihydroflavonols to form the requisite alcohol for ANS activity. ANS oxidizes this substrate, yielding anthocyanidins, or proanthocyanidins (Saito et al., 1999). Two additional 2-ODDs have been detected in saxifrage (*Chrysosplenium americanum)* and sweet basil (*Ocimum basilicum)*. Flavonol 6-hydroxylase from saxifrage preferentially hydroxylates the C-6 position of trimethylquercetin to yield 6-hydroxy-trimethylquercetin (Figure 5B; Anzellotti and Ibrahim, 2004). The enzyme from sweet basil catalyzes the *O*-demethylation of gardenin B to nevadensin in the presence of 2-ODD co-factors and reaction conditions, however, the corresponding gene has not been isolated (Figure 5C; Berim and Gang, 2013).

### Benzylisoquinoline alkaloid biosynthesis

Benzylisoquinoline alkaloids (BIAs) are a group of plant specialized metabolites derived from the amino acid tyrosine. The most important source of BIAs is opium poppy (*Papaver somniferum*), which has the exclusive ability to synthesize several medicinal compounds including the narcotic analgesics morphine and codeine, the vasodilator papaverine, the cough suppressant and potential anti-cancer drug noscapine, and the precursor to several semi-synthetic opiates thebaine. Opium poppy remains the sole commercial source for these compounds. Other BIAs include sanguinarine and magnoflorine, which are thought to function as phytoalexins (Weiss et al., 2006).

The committed step leading to BIAs is catalyzed by norcoclaurine synthase (NCS), which catalyzes the condensation of two tyrosine derivates to form the central intermediate (*S*)-norcoclaurine (Samanani et al., 2004). (*S*)-Norcoclaurine is converted to the key branch-point intermediate (*S*)-reticuline, from which nearly all BIA subclasses are derived (Ziegler and Facchini, 2008). Recently, a 2-ODD-like enzyme from *Coptis japonica* (Minami et al., 2007) was reported to exhibit NCS activity, however, the results remain questionable (Lee and Facchini, 2010). Interestingly, this 2-ODD was not used in an engineered microbial system for the production of (*S*)-reticuline, in favor of an entirely unrelated enzyme (Nakagawa et al., 2011).

**Figure 6:**
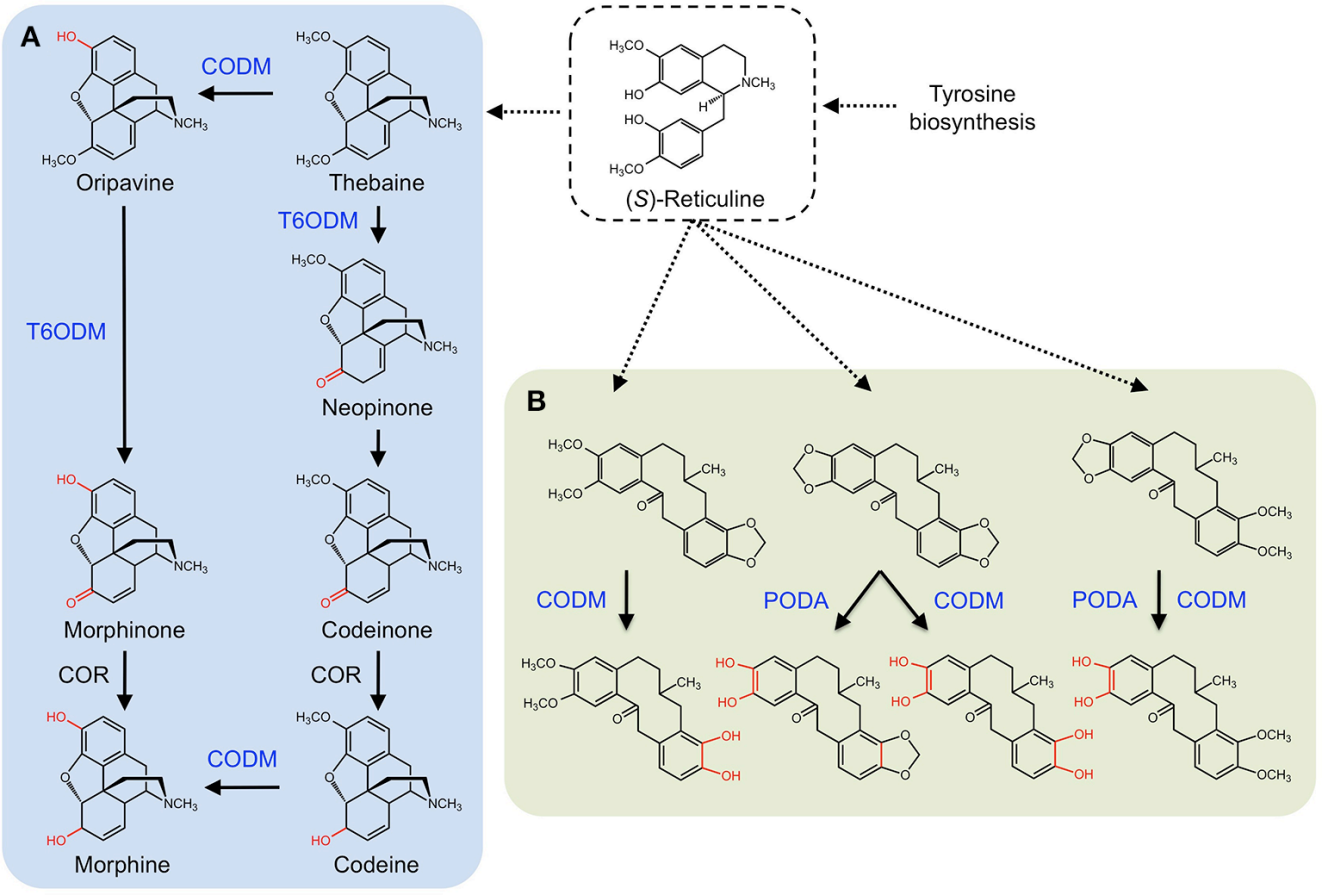
**Benzylisoquinoline alkaloid biosynthesis in *Papaver somniferum***. (*S*)-reticuline is an important branch-point intermediate leading to many alkaloids including the morphinans **(A)** and protopines **(B)**. **(A)** CODM and T6ODM are responsible for the *O*-demethylation steps leading to morphine. **(B)** CODM and PODA catalyze the *O*-demethylations and *O,O*-demethylenation of protopines.

CYPs were long considered responsible for the late *O-demethylation* steps in the branch pathway converting (*S*)-reticuline to morphine in opium poppy owing to the activity of human CYP2D6, which catalyzes the reactions (Grobe et al., 2009). Using an opium poppy DNA microarray to compare the transcriptomes of high-morphine and morphine-free chemotypes, a gene encoding a 2-ODD was identified (Hagel and Facchini, 2010). The recombinant protein catalyzed the 6-*O*-demethylation of thebaine and oripavine, and the enzyme was named thebaine 6-*O*-demethylase (T6ODM) (Figure 6A). Two related 2-ODDs were identified in opium poppy. One, codeine *O*-demethylase (CODM), showed strict 3-*O*-demethylation activity with thebaine and codeine (Hagel and Facchini, 2010).

Subsequently, broader metabolite functions were detected for T6ODM and CODM in opium poppy, and a role for the previously uncharacterized 2-ODD was determined (Farrow and Facchini, 2013). Surprisingly, T6ODM, CODM, and protopine *O*-dealkylase (PODA) catalyze the *O*-demethylation of several BIAs with *O*-linked methyl groups. Moreover, CODM, and PODA catalyze the *O,O*-demethylenation of protopine and/or protoberberine alkaloids (Figure 6B). The *O,O*-demethylenation activity is the first associated with a plant enzyme. Previously, only mammalian CYPs were shown to *O,O*-demethylenate certain compounds containing methylenedioxy bridges. Virus-induced gene silencing (VIGS) in opium poppy plants confirmed not only the participation of T6ODM and CODM in protopine metabolism, but unexpectedly highlighted a possible role in the regulation of sanguinarine levels (Farrow and Facchini, 2013). Sanguinarine and protopine are widely distributed in plants suggesting a possible ancestral function for 2-ODDs, from which opiate *O*-demethylation evolved. Interestingly, domain-swapping of non-conserved regions of T6ODM and CODM (Runguphan et al., 2012) resulted in a mutant capable of *O*-demethylating codeine, but not thebaine (Figure 1).

### Glucosinolate biosynthesis

**Figure 7:**
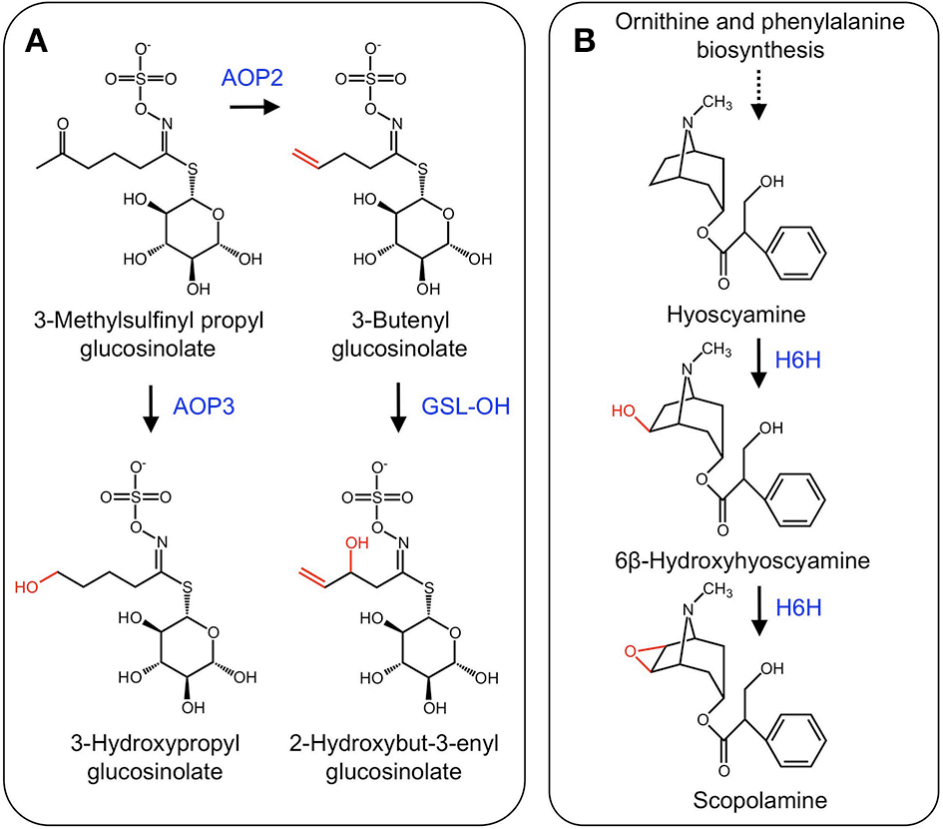
**(A)** Side-chain modification of glucosinolates catalyzed by AOP2, AOP3, and GSL-OH. **(B)** Hydroxylation of hyoscyamine to 6β-hydroxyhyoscyamine and subsequent epoxidation to scopolamine by H6H.

Glucosinolates are nitrogenous and sulfur-containing compounds found mainly in the order Brassicales and known for their pungent taste in foods such as mustard greens (*Brassica juncea)* and horseradish (*Armoracia rusticana*). Like other plant specialized metabolites, glucosinolates serve as protective agents or insect attractants. The biosynthesis of glucosinolates begins with one of three amino acid substrates, which are oxidized by CYPs to form an oxime. The introduction of a sulfur atom through *aci*-nitro and *S*-alkyl-thiohydroximate intermediates and subsequent addition of a glucose moiety yield the glucosinolate scaffold (Halkier and Du, 1997). Modification of the glucosinolate side chain occurs via several enzyme types, including methylthioalkyl-malate synthases that control the carbon chain length of the final glucosinolate structure, a family of glucosinolate flavin monooxidases that oxidize methylthioalkyl glucosinolates to their corresponding methylsulfinylalkyl derivatives, and a family of 2-ODD that modify the methylsulfinylalkyl glucosinolate side chains to alkenyl and hydroxyalkyl glucosinolates (Halkier and Du, 1997; Hansen et al., 2008). Discovery of a small gene cluster encoding 2-ODDs was achieved using a fine-scale mapping approach. Two genes encode enzymes (AOP2 and AOP3) involved in glucosinolate side chain modification. AOP2 catalyzes the conversion of 3-methylsulfinylpropyl to the allyl glucosinolate, and 4-methylsulfinylbutyl to the 3-butenyl glucosinolate, whereas AOP3 catalyzes the transformation of the 3-methylsulfinylpropyl glucosinolate to 3-hydroxypropyl glucosinolate (Kliebenstein et al., 2001, Figure 7A). Recently, a third 2-ODD (GSL-OH) was discovered that operates immediately after AOP2. Specifically, GSL-OH catalyzes the 2-hydroxylation of but-3-enyl glucosinolate to 2-hydroxybut-3-enyl glucosinolate (Figure 7A). Interestingly, GSL-OH was apparently recruited from a different 2-ODD clade, and yields a reaction product that is more toxic to pests than upstream intermediates. Additionally, 2-hydroxybut-3-enyl glucosinolate is a major source of bitter flavor in cruciferous vegetables. Tissue disruption causes hydrolysis of 2-hydroxybut-3-enyl glucosinolate to a goitrin, which precludes the use of cruciferous crops as a cattle feed. In contrast, conversion of 2-hydroxybut-3-enyl glucosinolate to a nitrile yields a compound that stimulates antioxidant pathways in humans (Hansen et al., 2008).

### Tropane alkaloid biosynthesis

The tropane alkaloids (TAs) occur mainly in the Solanaceae family, but are scattered throughout other plant species (Jirschitzka et al., 2012). TAs are a hallucinogenic and toxic group of plant specialized metabolites, the most notorious of which is cocaine (Docimo et al., 2012). Hyoscyamine and scopolamine that are the two most pervasive TAs and are used for the treatment of several ailments (Li et al., 2012). TA biosynthesis begins with the amino acids L-ornithine or L-arginine, which are converted to putrescine. Several other conversions leading to hyoscyamine have been elucidated (Ziegler and Facchini, 2008). The final two-step conversion of hyoscyamine to the 6,7-epoxide scopolamine is catalyzed by the 2-ODD hyoscyamine 6β-hydroxylase (H6H) (Figure 7B) (Hashimoto and Yamada, 1986, 1987). Genes encoding H6H have been isolated from several solanaceous plants including *Atropa belladonna* (Li et al., 2012), *Hyoscyamus niger* (Matsuda et al., 1991)*, Atropa baetica* (El Jaber-Vazdekis et al., 2009), *Anisodus acutangulus* (Kai et al., 2007), *Anisodus tanguticus* (Liu et al., 2005), and *Datura metel L*. (Pramod et al., 2010).

### Monoterpene indole alkaloid biosynthesis

**Figure 8:**
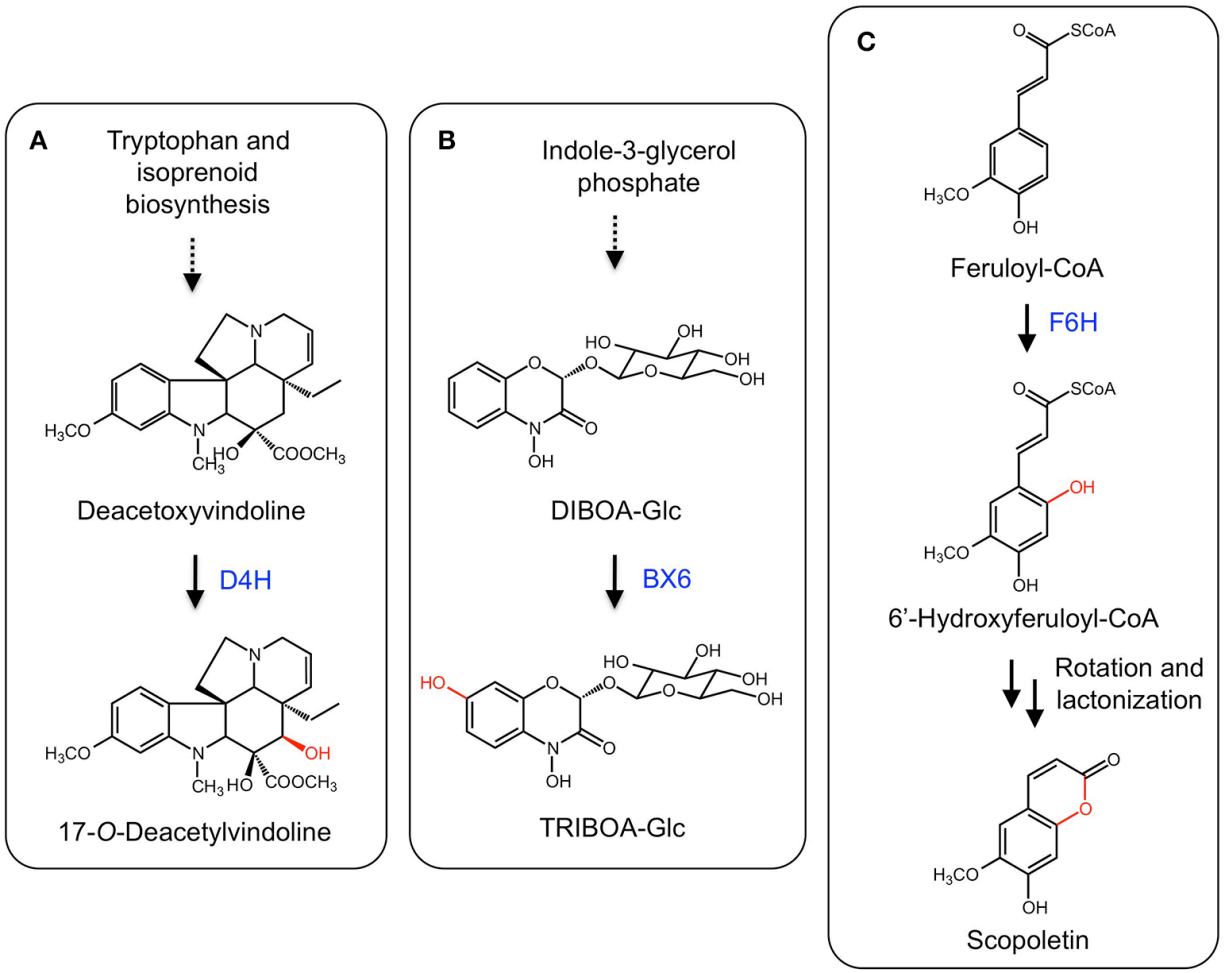
**(A)** Hydroxylation of deacetoxyvindoline by D4H leading to 17-*O*-deacetylvindoline. **(B)** B6X hydroxylates DIBOA-Glc to form TRIBOA-Glc. **(C)** Hydroxylation of feruoyl-CoA by F6H yielding scopoletin via spontaneous rotation and lactonization.

The monoterpene indole alkaloids (MIAs) are a class of plant specialized metabolites found mainly in members of the Apocynaceae, Loganiaceae, and Rubiaceae families. Several MIAs are medicinally important including the anticancer agents vinblastine, vincristine, and camptothecin, the anti-malarial drug quinine, and the antihypertensive compound ajmalicine (De Luca et al., 2014). In the biosynthetic pathway leading to vinblastine, vindoline is one of the monomeric components of the active dimer. The biosynthesis of vindoline in *Catharanthus roseus* involves the hydroxylation of desacetoxyvindoline to the immediate precursor of vindoline, deacetoxyvindoline, by the 2-ODD desdeoxyvindoline 4-hydroxylase (D4H) (Figure 8A; De Carolis et al., 1990; De Carolis and De Luca, 1993; Vazquez-Flota et al., 1997).

### Benzoxazinoid biosynthesis

Benzoxazinoids such as DIBOA and DIMBOA are allelophatic compounds mostly found in members of the Poaceae family, which includes maize (*Zea mays*), wheat (*Triticum aestivum*), and rye (*Secale cereale*) (Dick et al., 2012). Benzoxazinoids in grasses are derived from tryptophan and a number of enzyme types are involved in DIMBOA biosynthesis, including an indole-3-glycerol phosphate lyase, CYPs, an UDP-glycosyl transferase, an *O*-methyltransferase, and a 2-ODD. In maize, DIBOA-glucoside is hydroxylated into TRIBOA-glucoside by the 2-ODD BENZOXAZINLESS6 (BX6) (Figure 8B; Frey et al., 2003). BX6 was implicated by treating maize seedlings with the 2-ODD inhibitor prohexadion-Ca, which reduced the accumulation of DIMBOA. Diagnostic sequence motifs of known 2-ODDs showed that *BX6* was part of a DIBOA gene cluster. Transposon insertional mutants of *BX6* markedly reduced DIMBOA biosynthesis. Subsequent biochemical analyses confirmed the activity and specificity of BX6 for DIBOA-glucoside (Jonczyk et al., 2008).

### Coumarin biosynthesis

Coumarins are a group of common plant defense compounds derived from phenylpropanoid metabolism, partially following the pathway to lignin biosynthesis. The branch point involves the *ortho*-hydroxylation of cinnamates, which is critical for subsequent *trans-cis* isomerization and lactonization reactions completing the formation of the core 2H-1-benzopyran-2-one structure of coumarins. The *ortho*-hydroxylation of cinnamates was presumed to be catalyzed by a CYP based on the occurence of enzyme activity in chloroplastic fractions (Gestetner and Conn, 1974; Kai et al., 2008). However, a T-DNA mutation of an Arabidopsis gene (*F6′H1*) encoding a 2-ODD reduced scopoletin levels in roots. Two recombinant enzymes (F6′H1 and F6′H2) exhibited *ortho*-hydroxylation activity with relative specificity for feruloyl-CoA (Figure 8C). Whereas *F6′H1* was expressed, *F6′H2* transcript levels were low and a corresponding T-DNA mutant did not affect scopoletin accumulation (Kai et al., 2008). Similar 2-ODDs have been discovered from sweet potato (*Ipomoea batatas*; Matsumoto et al., 2012) and common rue (*Ruta graveolens*; Vialart et al., 2012). One sweet potato enzyme catalyzed the *ortho*-hydroxylation of feruoyl CoA, whereas a second from sweet potato and the common rue enzyme catalyzed the *ortho*-hydroxylation of *p*-coumaryl-CoA and feruoyl-CoA. The 2′-hydroxylase activity on *p*-comaryl CoA yields umbelliferone.

### Mugineic acid biosynthesis

**Figure 9:**
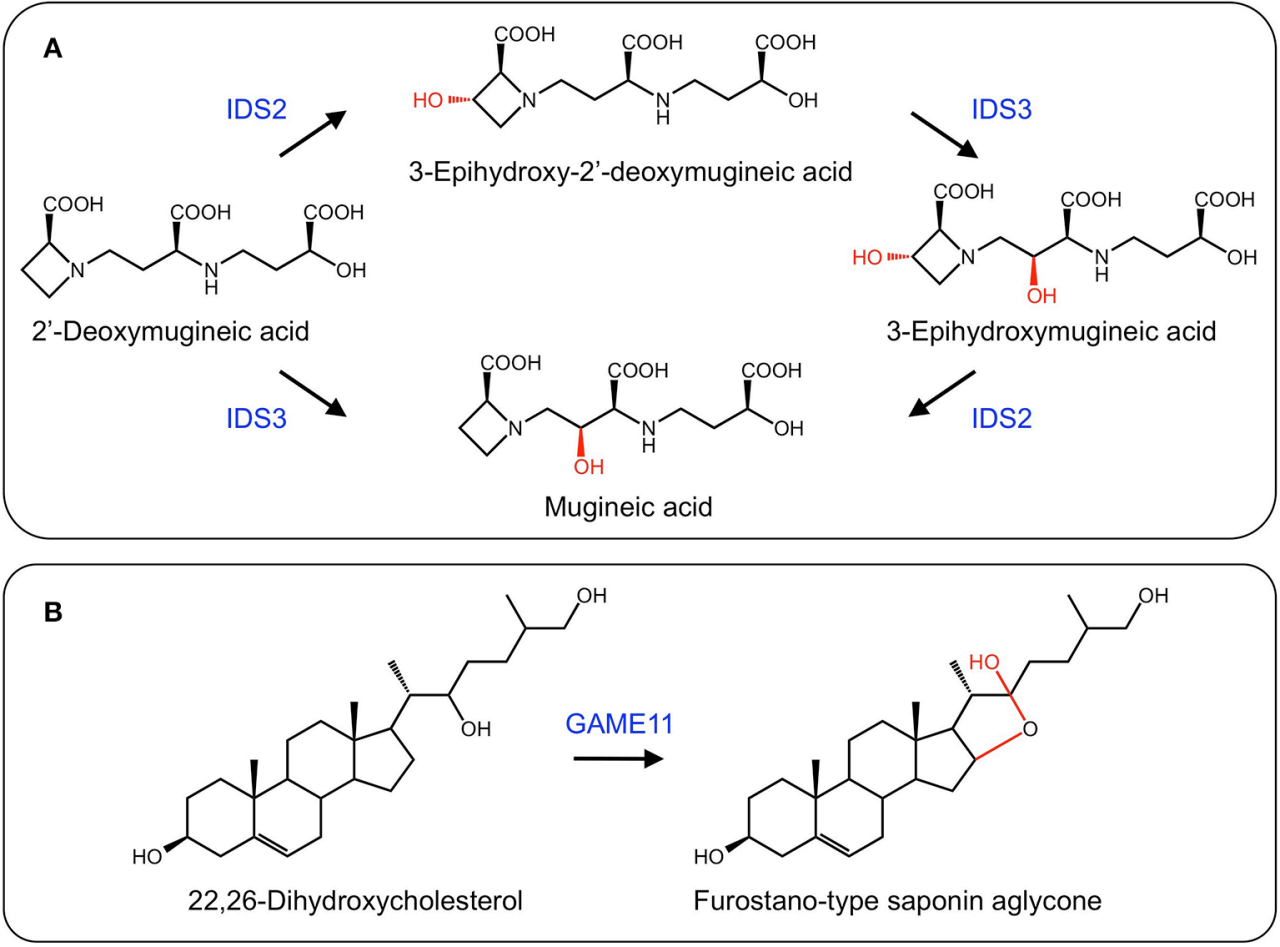
**(A)** Formation of iron scavenging mugineic acids by IDS2 and IDS3 (Nakanishi et al., 2000). **(B)** Proposed activity of GAME11 in steroidal glycoalkaloid biosynthesis.

In response to iron deficiency, graminaceous plants such as barley (*Hordeum vulgare*) and rye (*Secale cereale*) synthesize mugineic acid phytosiderophores to chelate iron from the soil. Mugineic acid biosynthesis begins with the conversion of *S-adenosyl-*L*-methionine* to 2′-deoxymugineic acid via nicotianamine synthase, nicotianamine aminotransferase, and a reductase. 2′-Deoxymugineic oxidation by two 2-ODDs, IDS2 and IDS3, yields 3-*epi*-hydroxy-deoxy mugineic acid and mugineic acid, respectively. Further oxidation of these compounds by the same enzymes yield *epi*-hydroxymugineic acid (Figure 9A) (Nakanishi et al., 1993, 2000; Okumura et al., 1994).

### Steroidal glycoalkaloid biosynthesis

Steroidal glycoalkaloids (SGs) are produced in solanaceous plants such as potato (*Solanum tuberosum*) and tomato (*Solanum lycopersicum*), and have negative health effects on humans. Several SG biosynthetic genes are clustered in the potato and tomato genomes, with others found on a duplicated region (Itkin et al., 2013). A gene encoding a 2-ODD, *GAME11*, occurs in the cluster and has been associated with steroidal glycoalkaloid biosynthesis. Using VIGS, suppression of *GAME11* resulted in a substantial decrease in α-tomatine and an increase in steroidal saponins suggesting that GAME11 competes for substrate with saponin biosynthetic enzymes. Currently, GAME11 is thought to hydroxylate 22,26-dihydroxycholesterol and assist in ring closure (Figure 9B). Interestingly, silencing *GAME4* led to a dramatic decrease in α-tomatine in both potato tubers and tomato fruit without pleiotropic effects, providing a means to eliminate these anti-nutritional compounds (Itkin et al., 2013).

## Evolution of 2-oxoglutarate/FE(II)-dependent dioxygenases

Analysis of 2-ODDs from green algae to angiosperms provided an interesting perspective on the evolution of plant 2-ODDs (Kawai et al., 2014). The conservation of genes responsible for DNA repair (AlkB), post-translational modification (P4H), and control of epigenetics (Jmj) across all taxa is attributed to essential roles in core metabolism. Such functionally diverse enzymes occur in distinct phylogenetic clades. Exceptional functional diversity is also evident among 2-ODDs involved in higher plant specialized metabolism, which also form a separate phylogenetic clade. Algae exhibit the fewest 2-ODDs involved in specialized metabolism and the number increases along evolutionary lines bryophytes to angiosperms. As a result of new environmental stresses associated with the migration to land, extensive duplication and functional diversification of genes encoding 2-ODDs allowed the formation of many new specialized metabolites (Kawai et al., 2014).

## Conclusions

We have highlighted the diversity of conversions catalyzed by 2-ODDs in plant metabolism. In primary metabolism, 2-ODDs have established roles in DNA repair, epigenetics, post-translational modification, and plant growth regulator activation and catabolism. In specialized metabolism, 2-ODDs participate in numerous pathways, and display as much functional diversity as CYPs.

### Conflict of interest statement

The authors declare that the research was conducted in the absence of any commercial or financial relationships that could be construed as a potential conflict of interest.
